# Brace yourselves: esports is coming

**DOI:** 10.17159/2078-516X/2020/v32i1a7596

**Published:** 2020-01-01

**Authors:** C Kemp, P R Pienaar, D E Rae

**Affiliations:** 1 Health through Physical Activity, Lifestyle and Sport Research Centre & Division of Exercise Science and Sports Medicine, Department of Human Biology, Faculty of Health Sciences, University of Cape Town, South Africa; 2 Department of Public and Occupational Health, Amsterdam Public Health Research Institute, VU University, Amsterdam UMC, Amsterdam, The Netherlands

**Keywords:** electronic sports, cybersports, computer gaming, professional gaming

## Abstract

**Background:**

Competitive gaming (or esports) is an emerging phenomenon with a field of over 454 million fans globally. Despite its tremendous popularity and commercial support, esports is not widely understood. It is also disregarded as a reputable or credible form of competition. The International Olympic Committee (IOC) contends that esports may be considered a sporting activity, but this is limited to the basis of its sedentary nature and poor governance.

**Discussion:**

These authors present evidence to inform and clarify misconceptions surrounding esports among the broader scientific community. They also encourage researchers to engage in further work into the phenomenon of competitive gaming with regard to health and performance, resulting in a better understanding of esports and guiding its development as a credible, competitive entity.

Esports is an emerging global phenomenon, defined as a type of organised professional video game competition. ^[1]^ Owing to the diverse nature of gaming, esports has also been described as a conglomeration of competitive video games. ^[1,2]^ Currently, it has a reach of 454 million fans with an estimated global market revenue of $1.1 billion (R16.5 billion). ^[2]^ On its current trajectory, with a compound annual growth rate of 14%, esports is expected to have a total global audience of 645 million fans by the year 2022. ^[2]^ While the global extent of esports currently appears to be overshadowed by mainstream sports, such as soccer, cricket and rugby, it certainly has garnered substantial public interest. ^[2]^ Compared to traditional US sports, esports dominates viewership figures in major US sports leagues, including their National Basketball Association, National Hockey League and Soccer Leagues. ^[3]^ In the US alone, esports viewership is estimated to reach 84 million by 2021, second only to the National Football League, with a projected 141 million viewers. ^[3]^ Moreover, these developments are not isolated to the US but extend across Europe and Asia. ^[2]^

Lucrative commercial partnerships have tracked the increased numbers of viewers, driving large-scale tournaments and events comparable with major traditional sports. Tournaments often take place in large, sold-out stadiums with multimillion-dollar prize pools. ^[4,5]^ This has been accompanied by both endemic sponsorships (e.g. computer and gaming brands) and non-endemic sponsorships (e.g. beverage brands) for teams and players. ^[2]^ Considering individual earnings, esports joins the ranks of traditional sports, with elite players earning as much as $3.1 million (R46.5 million) per single major event. ^[4]^ This has resulted in esports becoming a highly competitive environment, understandably placing pressure on these athletes to perform optimally.

## Discussion

### Training

Unlike traditional sports, esports is host to a dynamic competitive landscape. This comes in the form of an evolving “meta” (a colloquial term describing the current optimal playstyle) since the pace, style and features of the games change, following regular or seasonal gameplay updates. With a relatively short-lived career, marked by an early age of peak performance, ^[6]^ esports athletes must continually adapt their playstyles or risk losing their competitive status. It is reported that esports athletes may dedicate as much as 14 h per day to practices or matches, ^[5]^ risking potential burnout or fatigue. This has inspired some professional teams to incorporate wellness programmes into their schedules to improve player performance and career longevity. Elite esports organisations invest large amounts of money into residential gaming houses for players and coaches to enable them to follow strict training schedules over the competitive season. An article published in the Inven Global website (27 May 2018) details the typical training day schedule of esports athletes as follows: wake-up at 10 am followed by training from 11 am to 7 pm, after which players may stream games to their followers until bedtime (2 am). Although training schedules are similarly adopted among professional teams, they may change over in the non-competitive season. Nevertheless, these cumbersome routines demonstrate the discipline required to be proficient as an esports athlete. In fact, Michael Phelps honoured esports athletes at The Game Awards 2016 ceremony, saying: ‘There’s absolutely no question to me the level of skill, training and devotion it requires to become a professional gamer’.

### Challenges

Despite these developments, esports has been challenged in its widespread acceptance as a reputable form of competition. This follows persistent efforts by the esports industry to prove its legitimacy, which included it being used as an exemplar at the PyeongChang Olympic Winter Games in 2018. Esports partners have also since engaged in discussions with the International Olympic Committee (IOC) regarding its inclusion as a demonstration title at the 2024 Summer Olympic Games in Paris. Additionally, there has been continued deliberation surrounding its inclusivity as a sporting activity, particularly with regards to the sedentary nature of esports. ^[7]^ A statement made by the IOC at the 7^th^ Olympic Summit indicates that there is continued debate around the physicality of competitive gaming, such that the notion as to whether esports can be considered a sport in the traditional sense, requires further discussion.

According to Jenny et al. (2016), ^[5]^ the components of a sport must include the elements of play, rules, competition, be comprised of skill (as opposed to chance), have a broad following, including physical skills (i.e. ‘skilful and strategic use of one’s body’) and governance. Esports tick the first five elements, and few people would argue against the cognitive effort and skill required in gaming. Gamers utilise an array of cognitive processes (e.g. attention, reaction time, working memory) and fine motor skills (e.g. to use the mouse or keyboard); ^[6,8]^ all of which are central to their proficiency and require significant cognitive effort. The sticking point seems to be around the physicality of gaming. Indeed, sport should be viewed holistically, without necessarily prioritising athleticism. However, gaming is, at least for the most part, considered to be a sedentary activity. While there are sports like archery and shooting, which involve a limited degree of physical activity during competition, the physical training that these athletes undergo to successfully shoot is comparable to that of more traditional athletes. In this regard, current forms of video gaming simply do not compare. Given the increasing popularity of motion-based video games (or exergames) and virtual reality, which may involve more gross motor activity, perhaps esports will be viewed as a more credible sport in the future.

### Governance

Although esports mimics traditional sports in many ways (debate around physicality notwithstanding), arguably the greatest barrier regarding its inclusivity as a credible sport, is its lack of organisational structure. ^[7]^ This issue was raised by the IOC previously in 2017 in response to failed attempts of governance by various stakeholders to ensure the implementation of policies. The World Esports Association (WESA) is the purported representative body for esports players, teams and organisations. It is also an official signatory of the World Anti-Doping Agency. WESA inspires little confidence, however, to ensure or enforce compliance of regulatory practices. These should include: random drug testing, player transfers, drafting of player contracts, and arbitration. Since esports titles remain the intellectual property of their respective game publishers, there is at least some level of authority regarding the enforcement of standardised rules and penalties of misconduct. This may extend to tournament organisers, such as ESL formerly known as Electronic Sports League, who maintain these rules, as well as their own rules, including matters involving fraud and deception. Broad policy reform under the jurisdiction of a global esports authority, however, is necessary to allow unbiased control and enforcement of policies regarding the matters of players, organisations and tournament organisers.

## Conclusion

Esports continues to show record growth as a front-runner in the digital ecosystem. Despite esports being a highly competitive and commercially-invested form of competition, it currently remains segregated from the sporting community. A call to refine the current definition of sport to be more inclusive, without favouring physicality, ^[1]^ has initiated progress regarding the inclusivity of esports. In celebrating a new era in digital entertainment and sporting activity, researchers are encouraged to be objective and use the esports platform as an opportunity to extend health and performance research. Indeed, it is research of this nature which may drive the policy reform of esports. Furthermore, research should act to bridge the gap in the current understanding of esports and guide its development to become credible and broadly recognised. Perhaps its acceptance as a credible sport is not a matter of fact or opinion, but simply a matter of time.
